# Supplementary material for: MOJITOO: a fast and universal method for integration of multimodal single-cell data
Source: Bioinformatics. 2022 Jun 27;38(Suppl 1):i282–9. doi: 10.1093/bioinformatics/btac220 (PMC9235504; doi:10.1093/bioinformatics/btac220)
Supplement: btac220_Supplementary_Data [file btac220_supplementary_data.pdf]

Bioinformatics
doi.10.1093/bioinformatics/xxxxxx
Advance Access Publication Date: 2 April 2015
Applications Note

OXFORD

Genome analysis

Supplementary File - MOJITOO: a fast and universal method for integration of multimodal single cell data

Mingbo Cheng[1], **Zhijian Li**[1], **Ivan G. Costa**[1*]

[1] Institute for Computational Genomics, Joint Research Center for Computational Biomedicine, RWTH Aachen University Medical School, 52074 Aachen, Germany

*To whom correspondence should be addressed.

Abstract

© The Author . Published by Oxford University Press. All rights reserved. For permissions, please e-mail: journals.permissions@oup.com

Table S1. Benchmarking experiments on SKIN-SHARE data set (time elapsed in minutes). Of note LIGER could only be executed with up to 28,147 cells. We also include two versions of MOFA with the full input matrices (MOFA full) or with reduced input matrices (MOFA).

cells	DIABLO	LIGER	MOFA-Full	MOFA	MOJITOO	scAI	schema	Symph-Int	WNN
3000	2.00	0.65	1.65	0.46	0.42	5.69	2.12	0.46	0.51
6000	6.91	1.06	3.10	0.88	0.63	15.29	4.10	0.78	0.82
9000	13.33	1.63	4.58	1.14	0.88	33.37	4.84	1.10	1.17
12000	21.18	2.19	7.24	1.31	1.13	62.68	5.70	1.30	1.56
15000	28.52	2.59	11.20	1.60	1.37	121.74	6.98	1.59	1.92
18000	40.85	3.02	18.53	2.26	1.83	171.82	8.02	2.09	2.50
21000	53.88	3.61	34.51	2.58	2.08	249.61	9.08	2.43	2.90
24000	69.14	4.23	43.96	2.85	2.30	350.98	10.56	2.64	3.26
27000	89.49	4.58	52.47	3.19	2.56	485.13	11.79	2.95	3.68
30000	103.26	-	67.53	3.21	2.48	637.52	13.09	3.01	3.74

Table S2. Peak memory consumption in gigabytes. Of note LIGER could only be executed with up to 28,147 cells. We also include two versions of MOFA with the full input matrices (MOFA full) or with reduced input matrices (MOFA)

cells	DIABLO	LIGER	MOFA-Full	MOFA	MOJITOO	scAI	Schema	Symph-Int	WNN
3000	9.61	10.88	1.66	3.20	1.61	6.27	10.66	3.19	1.61
6000	10.87	9.99	2.37	4.76	2.11	10.65	11.41	4.74	2.11
9000	15.25	9.09	2.51	6.37	2.46	10.32	11.66	6.34	2.46
12000	20.34	12.90	3.90	7.95	3.28	14.26	11.68	7.91	2.89
15000	20.38	12.04	4.42	9.56	3.43	20.22	12.07	9.51	3.88
18000	35.06	16.80	6.58	9.09	4.31	26.36	12.39	9.09	4.09
21000	42.29	15.92	9.17	9.28	5.01	34.14	13.11	9.28	5.58
24000	50.61	21.85	12.49	8.86	4.85	43.27	13.29	8.86	5.84
27000	57.22	20.93	17.94	13.05	5.94	58.91	13.74	13.05	5.93
30000	78.30	-	22.47	13.09	6.34	75.92	14.28	13.09	6.79

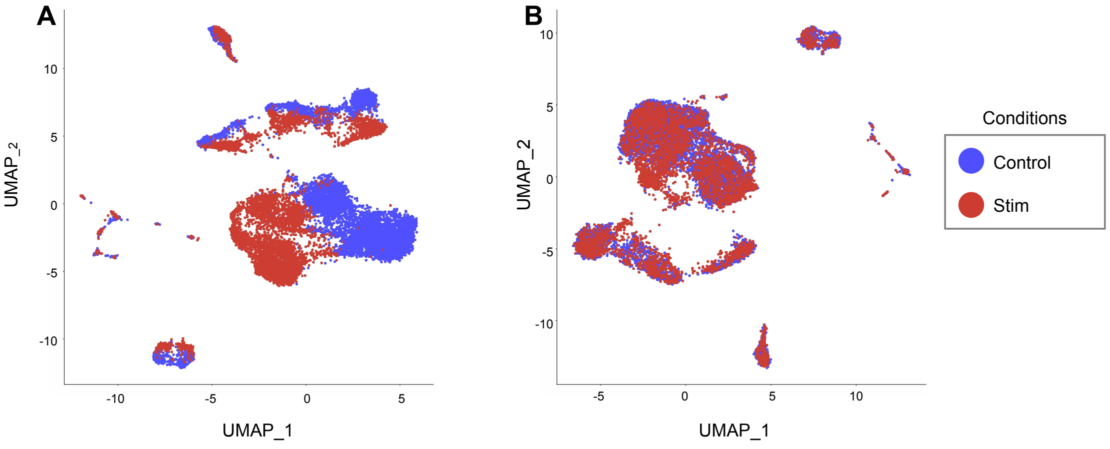

Fig. S1. UMAP contrasting MOJITOO latent spaces without (A,) and with (B,) Harmony batch corrections for control and stimulated samples of the PBMC-DOGMA data set.

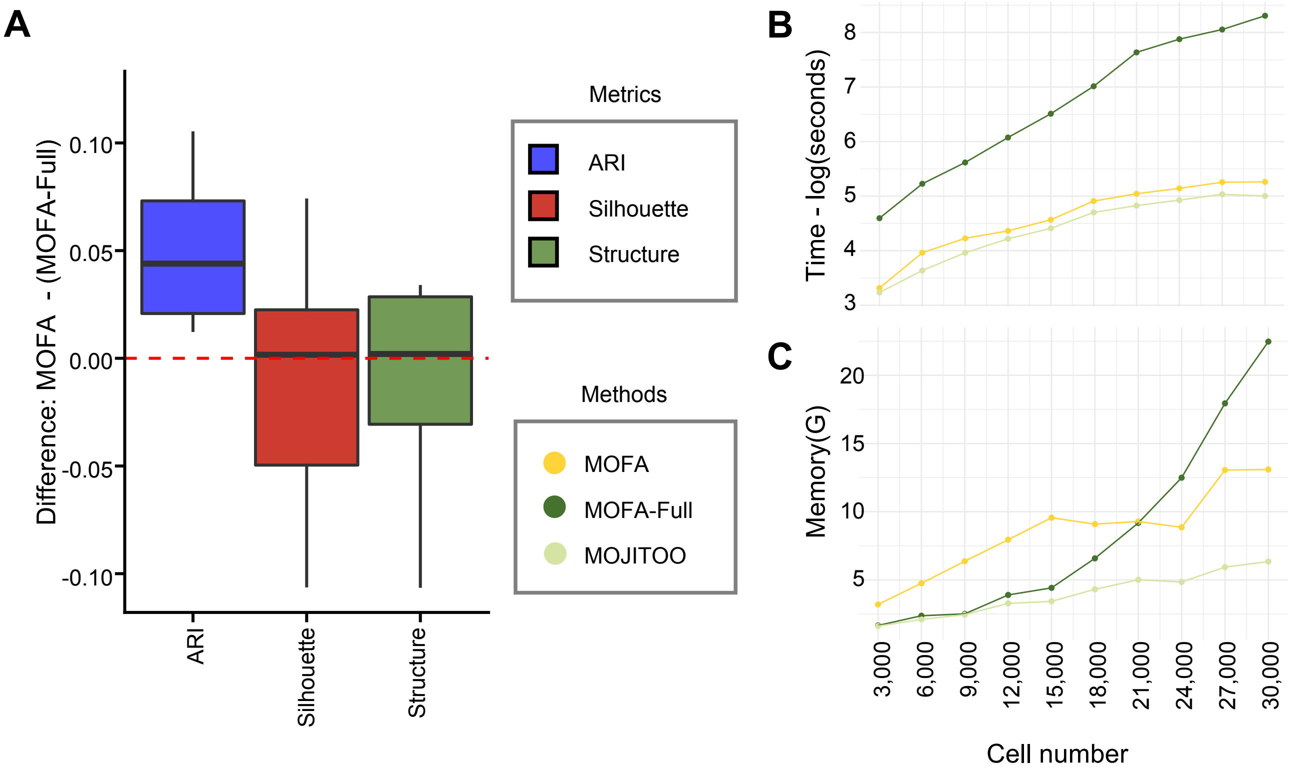

Fig. S2. A, Boxplots shows differences MOFA with dimension reduction and with raw count matrix (MOFA-full) for ARI, Sihouette, Structure in all benchmarking datasets. The red dash line indicate the difference is equal to 0. B, Line plots showing elapsed time (log of seconds) for MOFA with dimension reductions input, MOFA-full with raw count matrix and MOJITOO (y-axis). C,Line plots showing peak memory(Gigabytes) required by MOFA with dimension reductions input, MOFA with raw count matrix and MOJITOO (y-axis). In both B-C, the x-axis shows the number of cells used (randomly sampled) from the Skin-SHARE data.

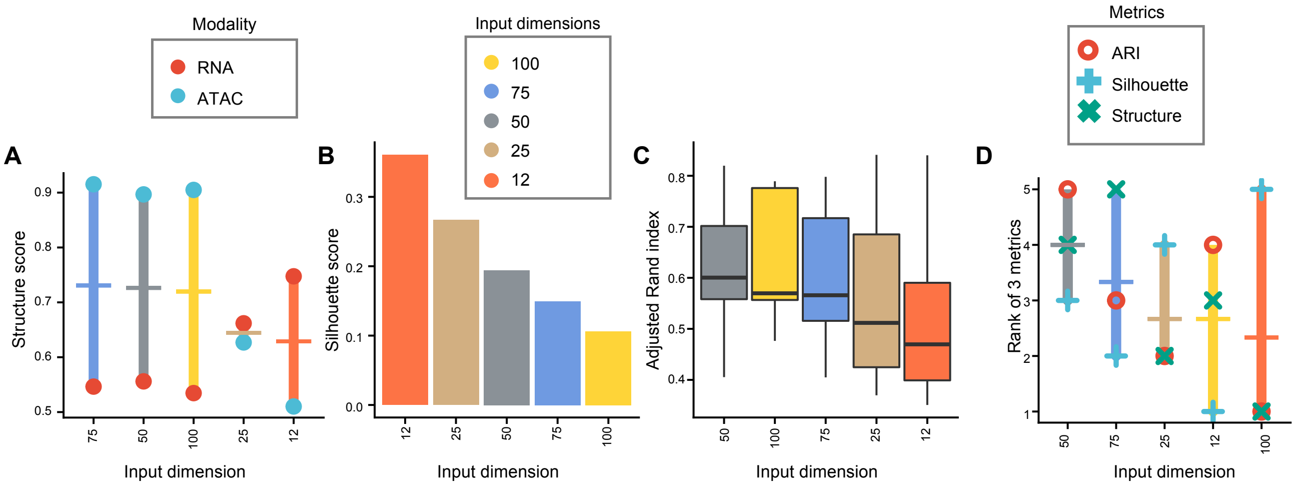

Fig. S3. MOJITOO applied to the PBMC-multiome data for distinct LSI and PCA dimensions.A, We shown the structure scores (y-axis) for distinct dimensions (x-axis). B, We shown the Silhouete scores (y-axis) for distinct dimensions (x-axis). C, We shown the distribution of Adjusted Rand score for distinct clustering results (resolution from 0.1 to 2.0) (y-axis) vs. number of components (x-axis). D, We shown the combined rank of the three previous metrics.

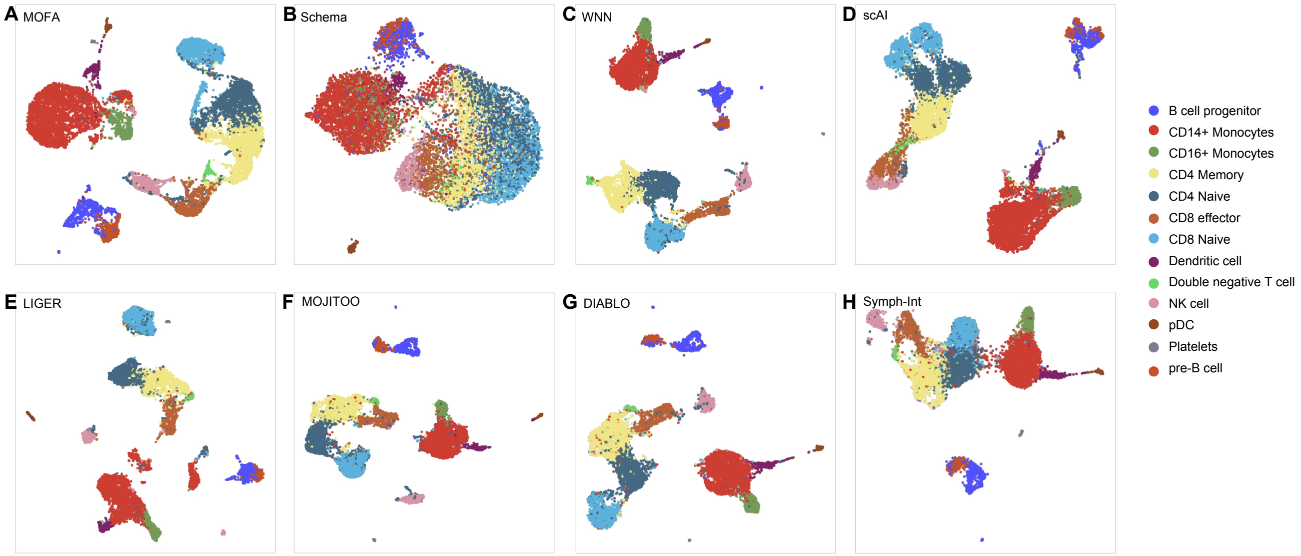

Fig. S4. A-H, UMAPs showing cell type distribution derived from integration methods on PBMC-multiome dataset.

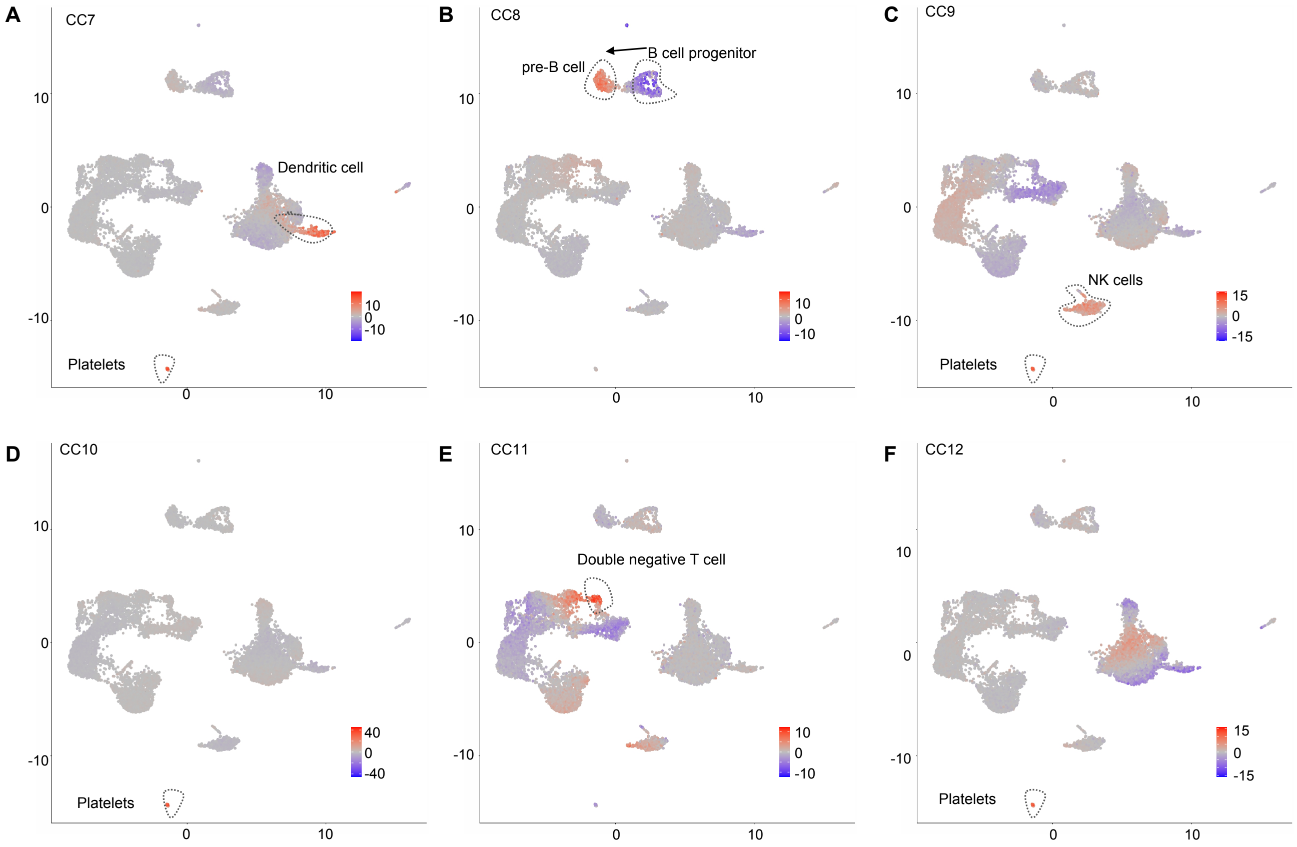

Fig. S5. A-F, UMAP with the scores of CC7 to CC12. We highlight major cell types associated to positive or negative CC scores and the arrow represents a potential differentiation process.

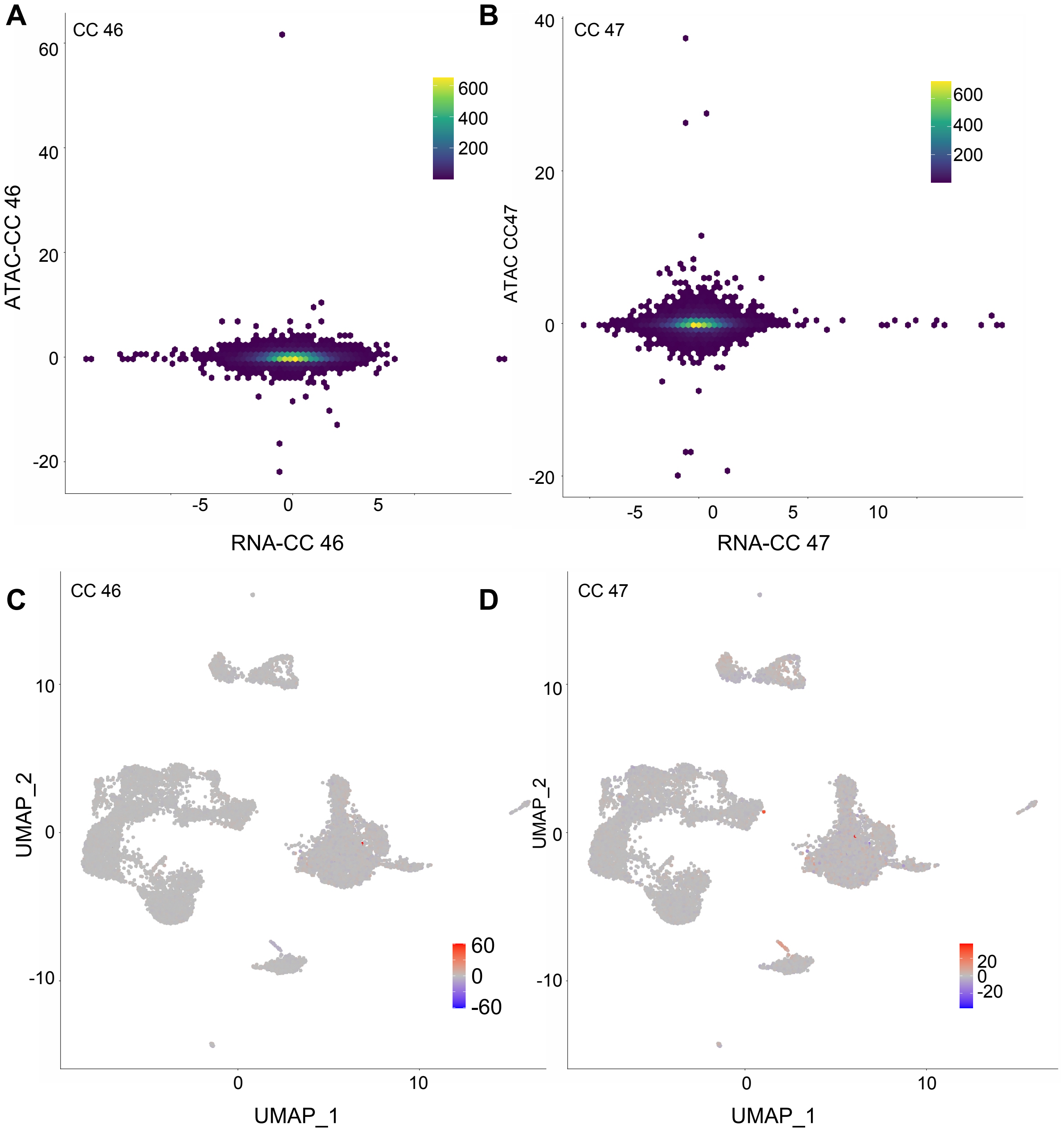

Fig. S6. A-B, Scatter plot comparing RNA and ATAC CCs in the PBMC-multiome data set. These CCs have low correlation and were filtered by MOJITOO C-D, Values of CC46 and CC47 in the UMAP space. Only a few cells have high CC scores, which support they are potential noise.
